# The Longitude-Latitude-Loop Used for Complex Bankart Lesion Repair: An All-Arthroscopic Technique

**DOI:** 10.1016/j.eats.2022.12.008

**Published:** 2023-03-27

**Authors:** Zhen-Ze Zheng, Chuan-Hai Zhou, Jin-Ming Zhang, Yuan-Hao Zhang, Min Zhou, Jing-Yi Hou, Rui Yang

**Affiliations:** Department of Orthopaedic Surgery, Sun Yat-sen Memorial Hospital, Sun Yat-sen University, Guangzhou, China

## Abstract

The most frequent operation for anterior shoulder instability is the arthroscopic Bankart repair, which has a positive outcome and a low rate of complications. A variety of restoration procedures have been reported to reconstruct labral height and reproduce a dynamic concavity–compression reaction. The longitude–latitude loop is a knotless high-strength suture method that simultaneously tightens the joint capsule in the warp and weft direction and resists tearing. The suture method is safe and reproducible. This study aimed to present a longitude–latitude loop suture for joint capsule labral complex repair during Bankart arthroscopy surgery.

Recurrent anterior shoulder dislocation is most prevalent in young and active individuals, and it can be caused by several anatomical diseases, ranging from simple anterior Bankart rips to more complicated capsular–labral tears, which can include both posterior and anterior problems.^1^ Most surgeons who treat traumatic recurrent anterior shoulder instability do use the Bankart repair. Since the Bankart repair was first performed in 1938, this approach has been improving and advancing both in terms of strengthening shoulder stability, ensuring long-term postprognostic outcomes, and reducing complications.^2^, ^3^, ^4^ Arthroscopic Bankart repair is the most commonly performed procedure worldwide for anterior shoulder instability. More specific Bankart repair methods, such as suturing methods and fixation methods, are also available, and studies have compared their advantages and disadvantages. What is the best and most stable method is still controversial and is lacking in proof. In addition, arthroscopic Bankart repair allows for a high rate of return to sport, with Memon et al.^5^ finding that 88% of patients returned to sport postoperatively.

Bankart repair procedures that reduce capsule volume have better clinical outcomes.^6^, ^7^, ^8^ Most current sutures have a latitudinal direction of tightening effect on the joint capsule, and some also can provide a longitudinal direction of tightening effect. Some technologies tighten the capsule laterally and act as an anticapsule tear. Meanwhile, one of the major goals of the Bankart repair process has been proven to be the restoration of labral height, thus enhancing the concavity stabilizing function by centering the humeral head in the glenoid.^9^, ^10^, ^11^, ^12^ A variety of techniques are available that can be classified as knotted or knotless, and articles have been written to demonstrate that knotless techniques are mechanically no lesser than knotted technologies.^13^ None of the technologies, however, combines these advantages at the same time. Therefore, in this Technical Note, we proposed a new suture technique, the longitude–latitude (LL) loop, which is a knotless method of Bankart repair that simultaneously tightens the joint capsule in the warp and weft direction and resists tearing. The LL loop is distinguished by its high capacity of latitudinal–longitudinal tightening of the joint capsule, self-anti-sawing tissue, knotless fixation, and a larger application market across different areas.

## Surgical Technique (With Video Illustration)

### Patient Positioning and Landmark Identification

**Fig 1:**
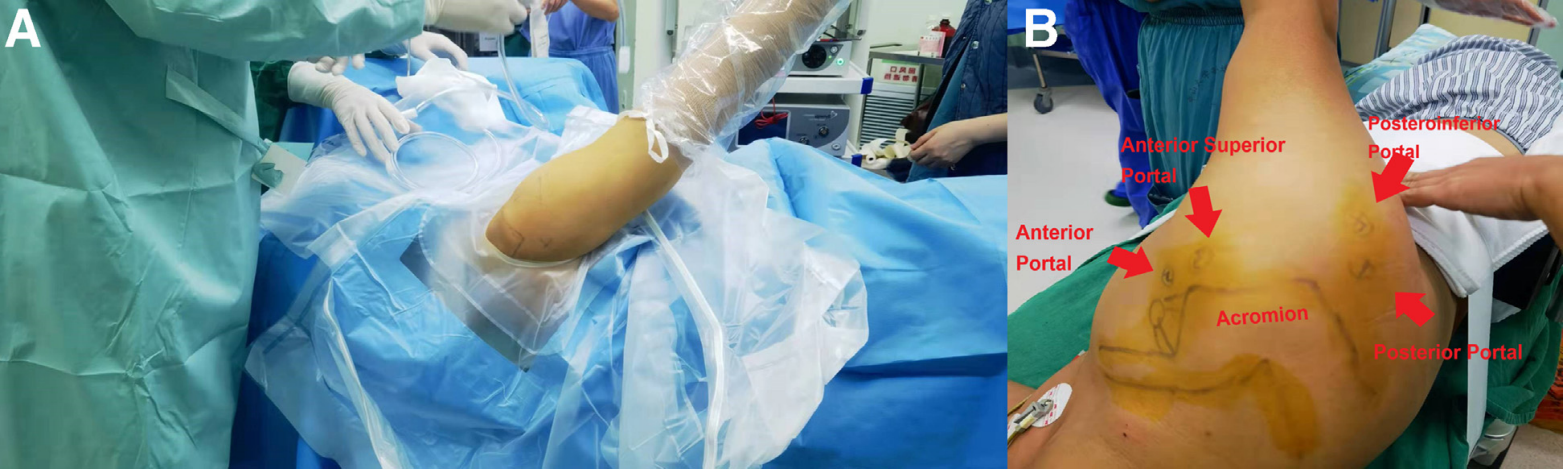
Identification of the patient’s location and landmarks. (A) The patient is positioned in a lateral decubitus posture with constant traction on the afflicted extremities. (B) The posterior, anterior, anterosuperior, and posteroinferior portals are delineated, as well as the bone markers.

The patient is positioned in the lateral decubitus position or the beach-chair position on the operating table after general anesthesia combined with a brachial plexus block, depending on the chief surgeon’s preference. The surgical shoulder is positioned at 70° forward flexion and 35°∼45° abduction in the lateral decubitus position. To gain more operating space in the glenohumeral joint, continuous traction is delivered via the ipsilateral afflicted upper extremity. The bone landmarks, as well as the posterior, anterior, anterosuperior, and posteroinferior portals, are recognized and indicated (Fig 1).

### Arthroscopic Examination and Bankart Pathology Identification

Arthroscopy is performed from the posterior portal, which is 2 cm posterior and 2 cm inferior to the posterior lateral angle of the acromion. Use a 30° arthroscope to enter the joint cavity and observe the Bankart lesion after filling the joint with water. A positive drive-through sign combined with significant anterosuperior dislocation of the humeral head can be observed. The cartilage and the lateral bone bed of the articular glenoid are thoroughly cleared and completely freshened with a shaver blade, in which the anterior portal is used at 11:00-1:00 o’clock, at 3:00-7:00 o’clock, and the anterosuperior portal is used at 1-3:00 o’clock. A more in-depth study of the lesions of the anterior and posterior capsule–labral complexes is undertaken. The area of the injured glenoid labrum is completely released using a rasp on the medial aspect of the subscapularis muscle belly to loosen the glenoid labrum capsule complex, which is manifested by the labrum floating on the articular surface (Video 1).

### The LL Loop Stitch Construction for Bankart

**Fig 2:**
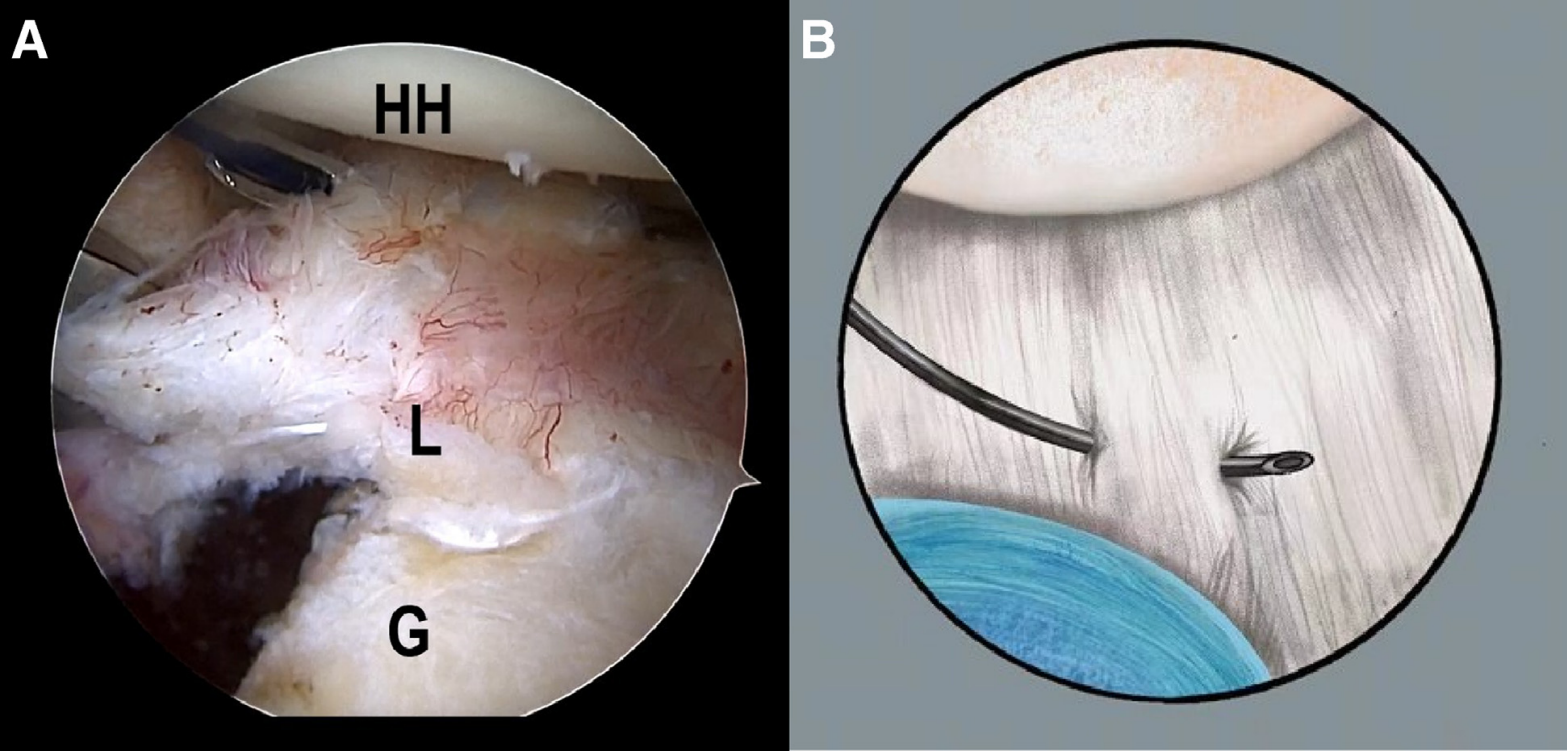
The patient is positioned in the lateral decubitus position. (A) An intra-articular arthroscopic image is shown of a left shoulder from the posterior viewing portal with a 30° arthroscope. A SutureLasso with a No. 0 PDS II suture is threaded outward through the joint capsule and into the joint after a parallel walk of 1 cm. (B) Illustration summarizing the corresponding step. (G, glenoid; HH, humeral head; L, labrum; PDS, polydioxanone.)

**Fig 3:**
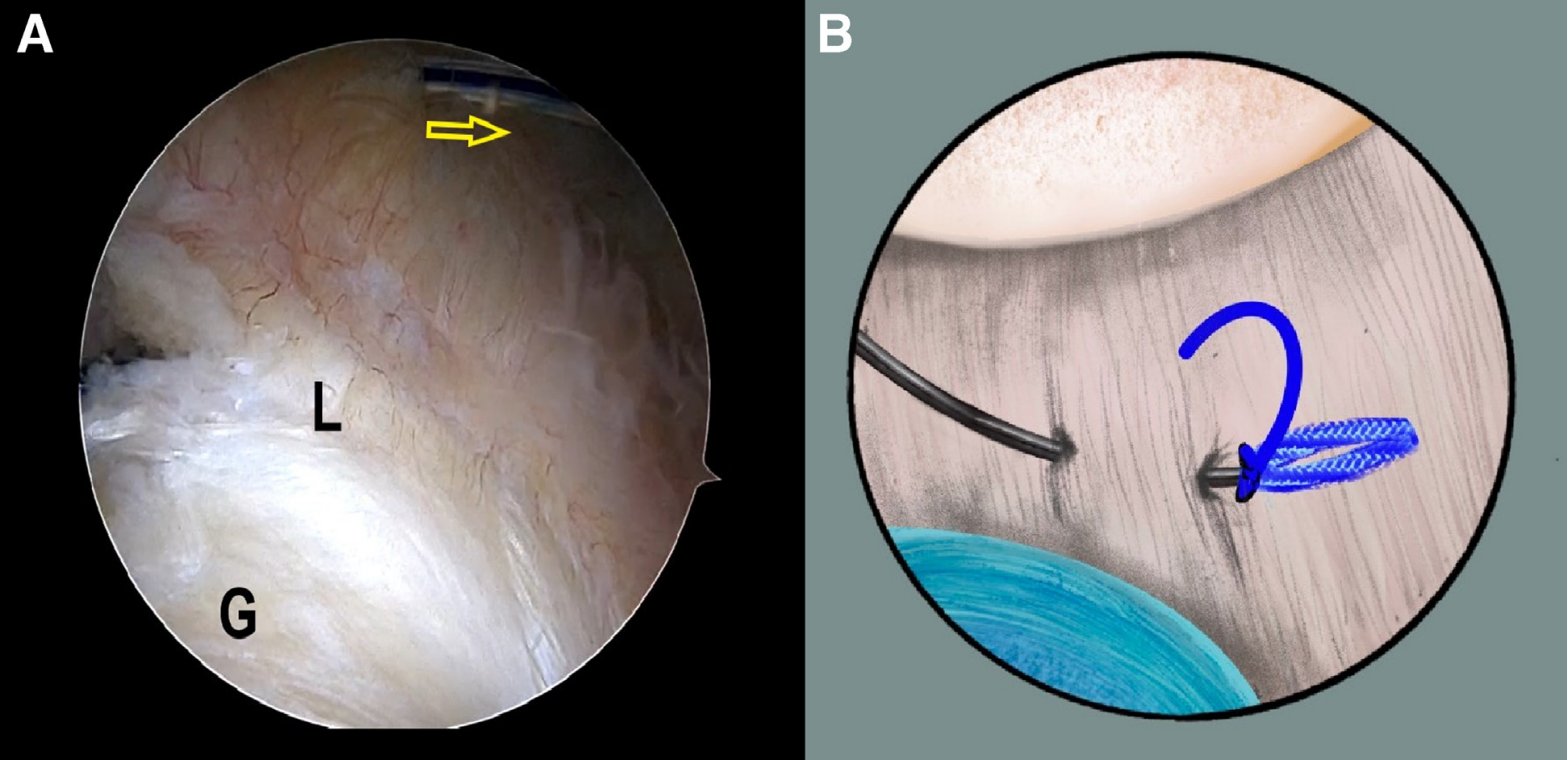
The patient is positioned in the lateral decubitus position. (A) An intra-articular arthroscopic image is shown of a left shoulder from the posterior viewing portal with a 30° arthroscope. A FiberWire is folded in half and pulled into the joint capsule via continuous traction by PDS after retrieving the SutureLasso and PDS inside the SutureLasso. (B) Illustration summarizing the corresponding step. (G, glenoid; L, labrum; PDS, polydioxanone.)

**Fig 4:**
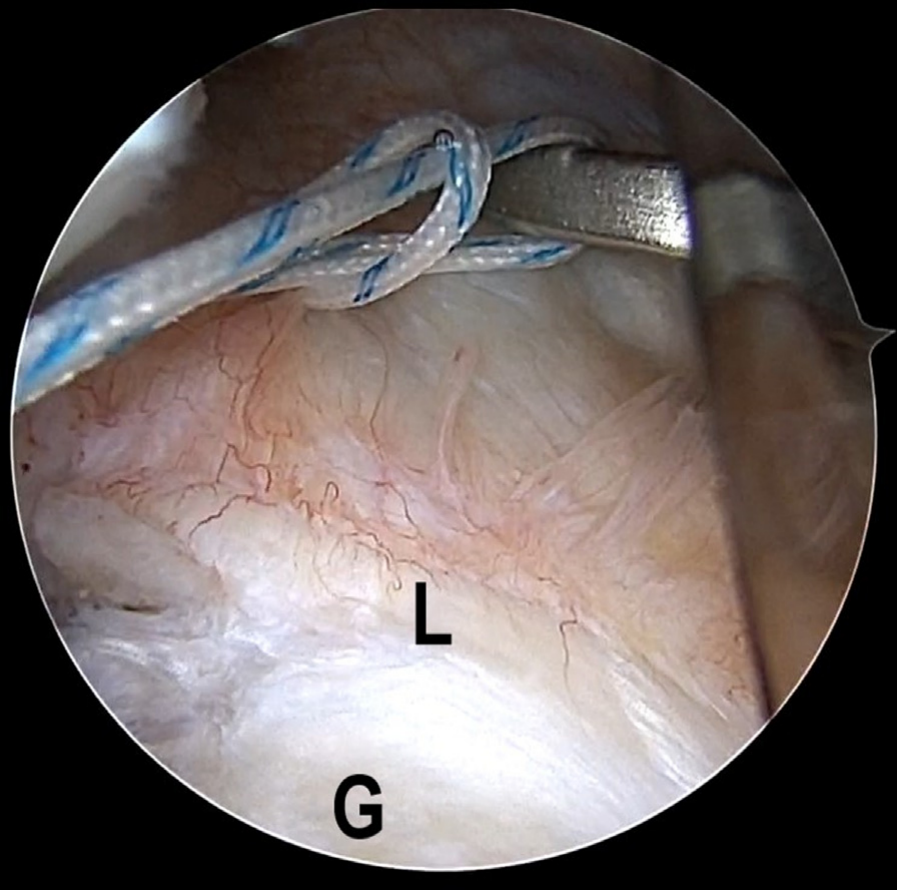
The patient is positioned in the lateral decubitus position. An intra-articular arthroscopic image is shown of a left shoulder from the posterior viewing portal with a 30° arthroscope. Two suture strands of the FiberWire are retrieved within the articular cavity and threaded into the loop to construct a lark’s head knot. (G, glenoid; L, labrum.)

Once the Bankart lesion is confirmed, the anchor placements are marked on the articular glenoid near the glenoid rim with the plasma vaporizing tip. A SutureLasso (Arthrex, Naples, FL), within a No. 0 PDS II (polydioxanone) suture (Ethicon [Johnson & Johnson], Somerville, NJ), is inserted into the joint capsule, through the posteroinferior portal. The first stitch starts with the 6:30-o'clock position, parallel to and 1 cm from the articular glenoid, and the SutureLasso is threaded outward through the joint capsule and into the joint after a parallel walk of 1 cm through the posteroinferior portal (Fig 2 A and B, Video 1). The end of the PDS is grasped out of the articular cavity through the anterior portal to tie an overhand knot on the middle point of a No. 2 FiberWire suture (Arthrex). Subsequently, the FiberWire is folded in half and pulled into the joint capsule via continuous traction by PDS, after retrieving the SutureLasso and PDS inside the SutureLasso (Fig 3 A and B, Video 1). Then, 2 suture strands of the FiberWire are retrieved within the articular cavity and threaded into the loop to construct a lark’s head knot (Fig 4, Video 1).

**Fig 5:**
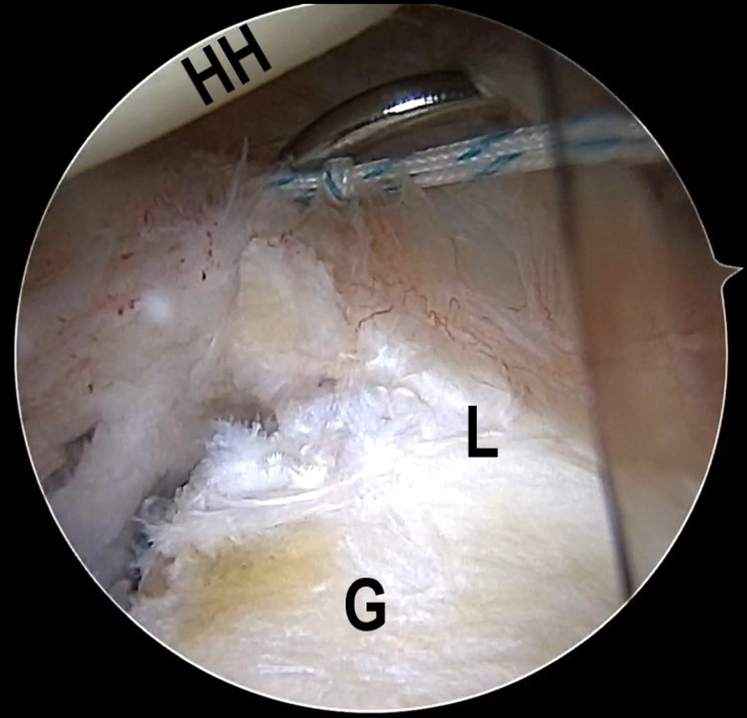
The patient is positioned in the lateral decubitus position. An intra-articular arthroscopic image is shown of a left shoulder from the posterior viewing portal with a 30° arthroscope. The tip of the SutureLasso is placed at a distance of 0.5 cm from the humeral side of the lark’s head knot. (G, glenoid; HH, humeral head; L, labrum.)

**Fig 6:**
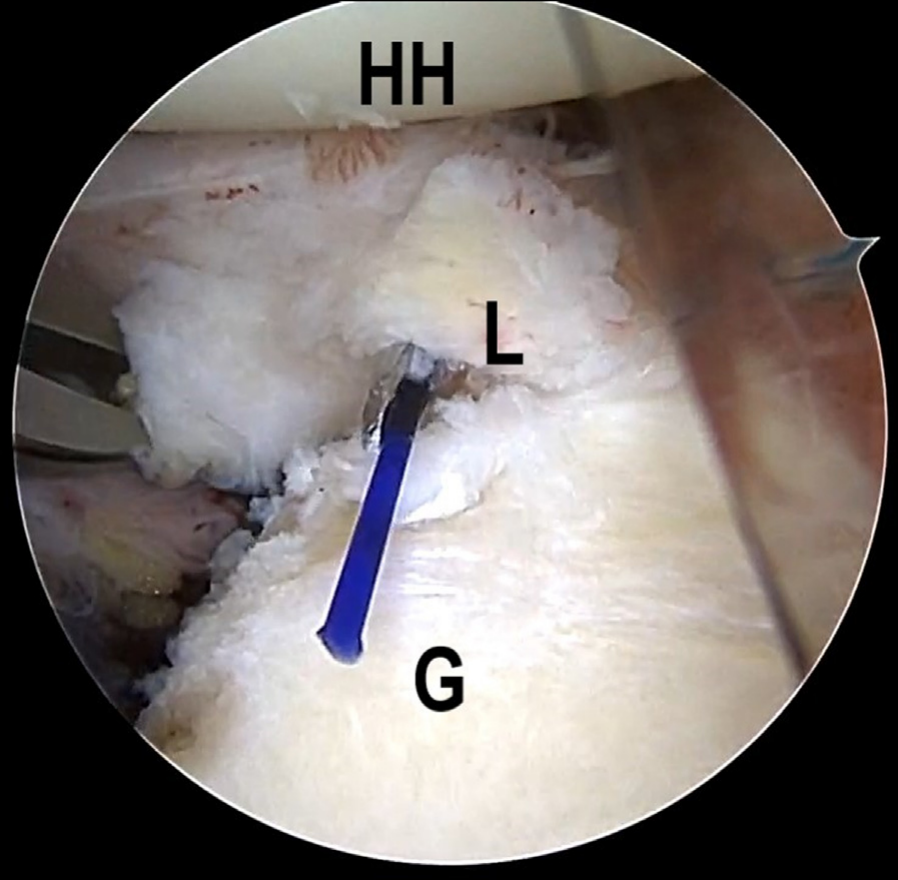
The patient is positioned in the lateral decubitus position. (A) An intra-articular arthroscopic image is shown of a left shoulder from the posterior viewing portal with a 30° arthroscope. The SutureLasso tip passes through the capsule tissue and exits through the tissue where the labrum and glenoid tear. (B) Illustration summarizing the corresponding step. (G, glenoid; HH, humeral head; L, labrum.)

**Fig 7:**
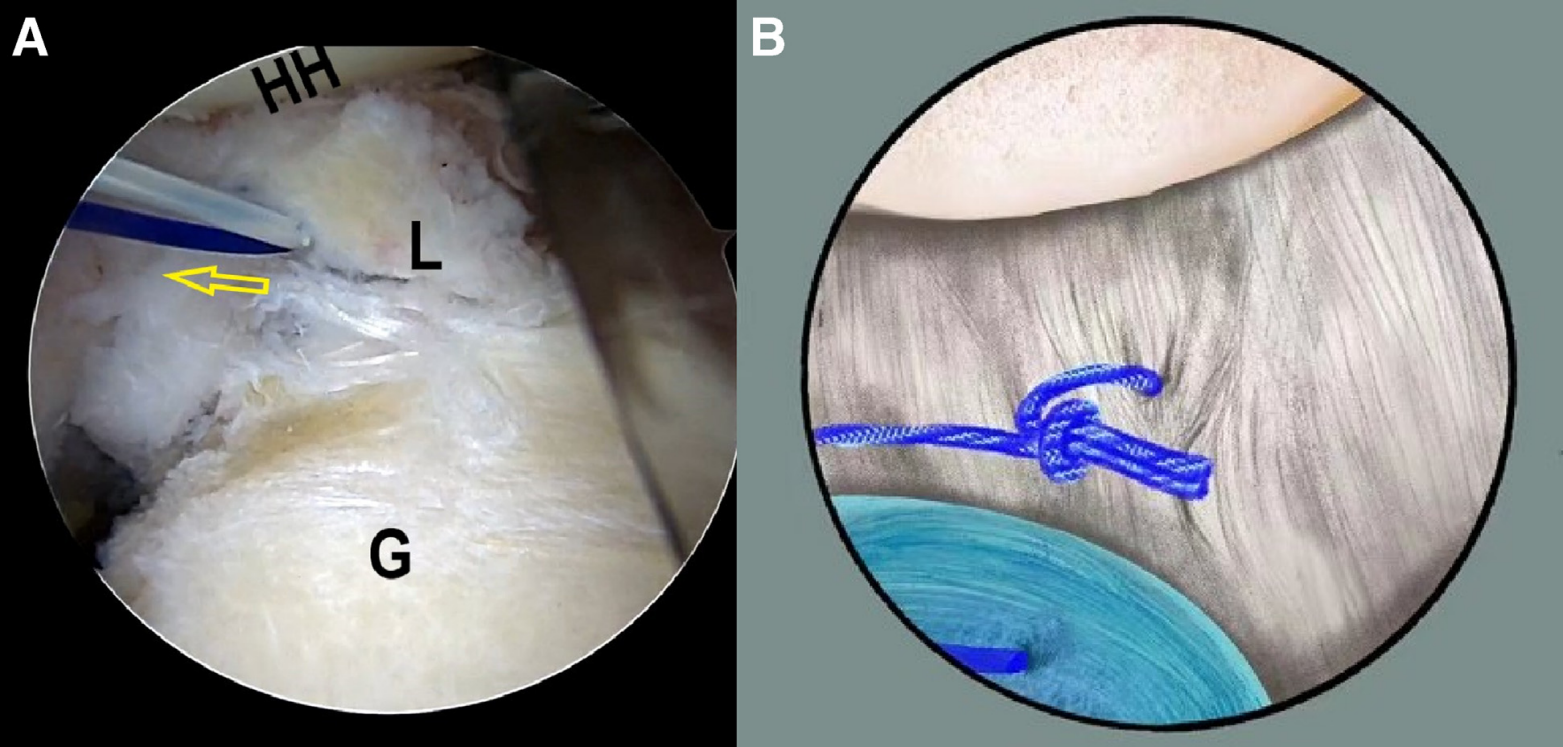
The patient is positioned in the lateral decubitus position. (A) An intra-articular arthroscopic image is shown of a left shoulder from the posterior viewing portal with a 30° arthroscope. The PDS is pulled out from the anterior portal side, with knot on one stand of FiberWire. (B) Illustration summarizing the corresponding step. (G, glenoid; HH, humeral head; L, labrum; PDS, polydioxanone.)

One of the 2 FiberWire strands is pulled out from the posteroinferior portal. Subsequently, the SutureLasso is inserted into the articular cavity through the posteroinferior portal carrying the PDS suture inside. The tip of the SutureLasso is placed at a distance of 0.5 cm from the humeral side of the lark’s head knot (Fig 5, Video 1). Then, the SutureLasso tip is passed through the capsule tissue and exits through the tissue where the labrum and glenoid tear to advance another PDS (Fig 6 A and B, Video 1). The end of the PDS is grasped out of the articular cavity through the anterior portal, whereas the SutureLasso is subsequently retrieved through the posteroinferior portal in sequence. Next, an overhand knot is tied on the FiberWire left in the posteroinferior portal cannula with the end of the PDS within the posteroinferior portal. Finally, the PDS is pulled out from the anterior portal side, helping to shuttle the FiberWire strand end through the capsule-labrum tissue (Fig 7 A and B, Video 1).

**Fig 8:**
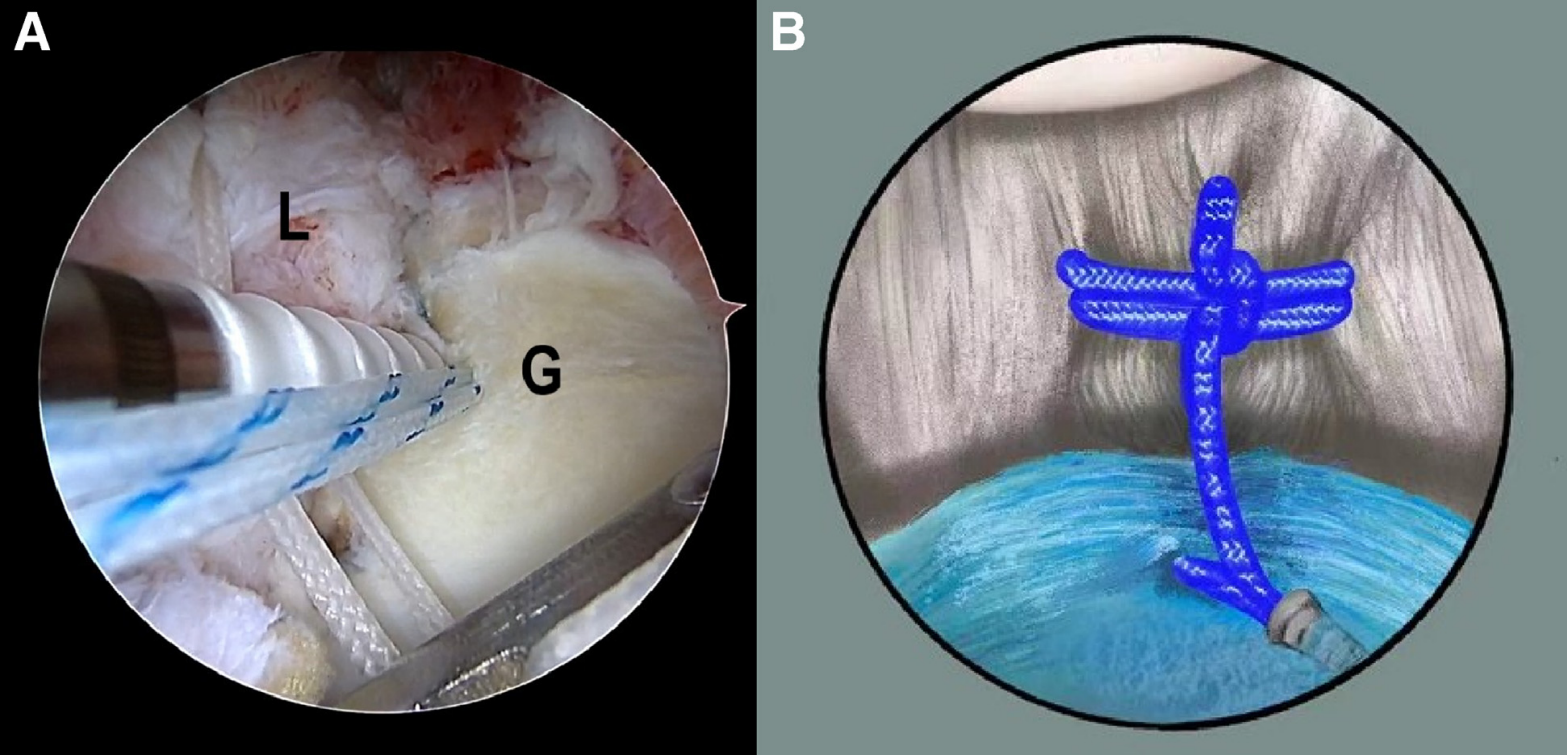
The patient is positioned in the lateral decubitus position. (A) An intra-articular arthroscopic image is shown of a left shoulder from the posterior viewing portal with a 30° arthroscope. After the initial fixation of the PushLock, the FiberWire strand of the labral side is tightened first to pull the labrum toward the glenoid surface and constrict the capsule in latitude. (B) Illustration summarizing the corresponding step. (G, glenoid; L, labrum.)

**Fig 9:**
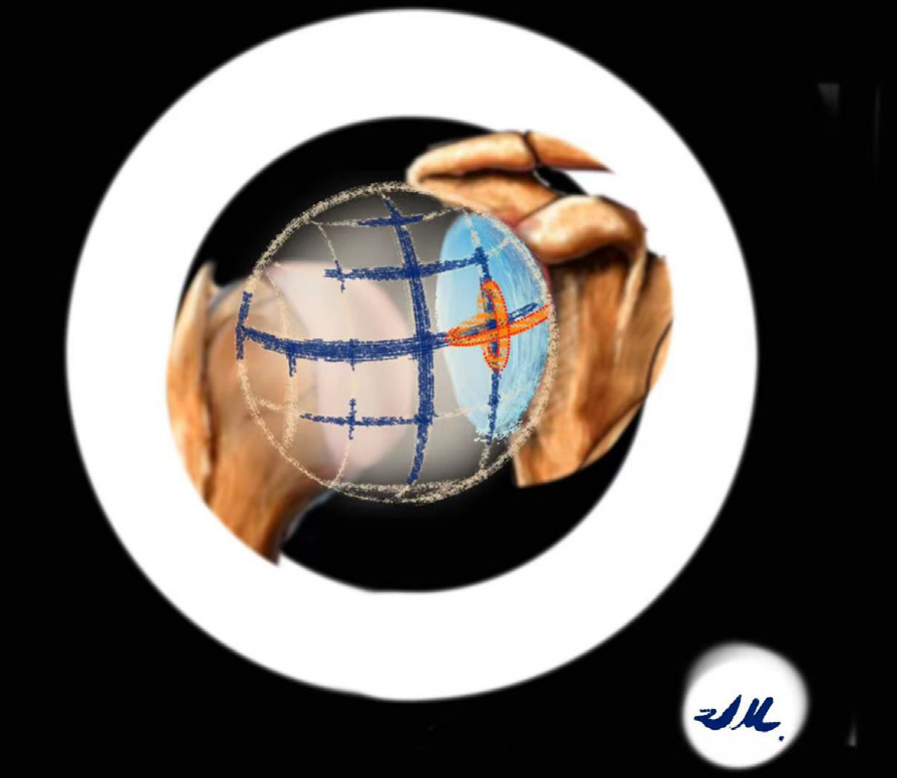
The loop is constructed, and it looks like the longitude–latitude line of the earth.

The procedure is repeated to construct the LL loop at the site of 5:20- to 4:30-, 4:20- to 3:30-, and 3:20- to 2:20-o'clock, respectively, with different colors (blue and white/black alternating). According to the LL loop location, a 2.9-mm hole is drilled at the 5:30-o’clock joint margin with the help of a 2.9-mm drill guide. The 2 FiberWire strands of the LL loop are passed through the eyelet of the BioComposite PushLock Anchor (Arthrex). (Fig 8 A and B, Video 1). In contrast, the other FiberWire strands of each loop are placed at the anterior portal outside the cannula and are kept pulling to help capsule-labrum reduction. After the initial fixation of the PushLock, the FiberWire strand of the labral side is tightened first to pull the labrum toward the glenoid surface and constrict the capsule in latitude. At the same time, the lark’s head knot will be tightened, either resulting in capsule constriction in longitude. Finally, the FiberWire strand of the glenoid side is tightened to constrict the capsule in longitude further. In the end, the PushLock is fixed firmly, while the height of the labral tissue is restored or even elevated to form the bump. Then, the residual suture is cut, and a novel, self-locking, and high-resistant loop configuration is constructed (Fig 9).

### Postoperative Rehabilitation

Patients undergo physical therapy in a sling for 4 to 6 weeks after the procedure, with external rotation limited to 10°. Patients need active movement of the affected wrist at full angle and the elbow at 90°-120° on the second day and continue for 6 weeks following the procedure. Limited passive joint mobility exercises are performed for the shoulder for 2-6 weeks. At 6 weeks, full range of active motion in all directions is allowed. Clinical follow-up is performed on patients after 3, 6, and 12 months.

## Discussion

Arthroscopic techniques for anterior shoulder instability continue to advance. There is a vast array of suture types and sizes, and therefore the surgeon must understand both the benefits and limitations of the implants to maximize a patient’s benefit from surgery.

This article describes a Bankart repair technique, the LL loop, a knotless, tear-resistant suture technique that tightens the capsule latitudinally and longitudinally and labrum reconstruction technique without any specialty sutures. Table 1 summarizes the technique-related tips and tricks, whereas Table 2 lists all of the benefits and drawbacks.

**Table 1:** Tips and Tricks

• The installation of a knotless anchor nail is made easier with a healthy anterior portal. • The anterior portal in the joint cavity should not be overly perpendicular or parallel to the articular glenoid. • To avoid aggravating cartilage and glenoid labral damage, clean the articular glenoid cartilage with the suction half-open. • To avoid insufficient glenoid labrum repositioning, the nail should be placed 0.5 cm above the longitude–latitude loop. • Completely release the glenoid labrum to the posterior lower section of the glenoid to avoid insufficient glenoid labrum height restoration.

**Table 2:** Benefits and Drawbacks

Benefits	Drawbacks
• This fully arthroscopic visualization technique can reduce the probability of intraoperative neurovascular injury and postoperative infection. • This technique simultaneously tightens the joint capsule latitudinally and longitudinally to reconstruct the glenoid labral complex. • The knot-free technique is free of mechanical defects and reduces residual thread reaction. • This technique acts as an anti-tear force in the latitudinal direction.	• New practice curves for operators. • More tedious and time-consuming to thread • Clinical follow-up data are scarce. • No experimental data on biomechanics and glenoid labrum height.

The LL loop tightens the joint capsule tissue in both the longitudinal and latitudinal directions, reducing the volume of the joint capsule, which has been shown to restore joint stability effectively. The loop that tightens the joint capsule laterally follows the fibers and does not cause much damage to the joint capsule. The capsule comprises fiber bundles with a radial orientation (coursing from the glenoid to the humerus), which are notably stronger and found in the deeper layer on the articular side, and fiber bundles with a circular orientation (coursing around the joint), which are largely in the superficial layer. The circular orientation of the fiber bundles is dominating in the superior section, but the radial components definitely prevail in the anteroinferior part.^14^ Tightening the joint capsule laterally not only reduces the distance between the humeral head and the glenoid, increasing joint stability, but also helps in raising the height of the glenoid labrum.

It has been demonstrated that one of the main objectives of the Bankart repair procedure is to restore the labral height, which is in the case of a LL loop mainly dependent on the latitude loop. It covers more labral complex tissue, strengthens the glenohumeral joint’s stability, and improves the labrum’s anatomical recovery. The creation of a new depression requires the restoration of labral height to support the glenohumeral joint. The labrum height following this procedure is no lower than after a simple suture or modified Mason–Allen, according to experienced arthroscopic users. The contact pressures exerted on the regenerated articular capsule tissue over the glenohumeral joint should be improved as a result of this.

As Wu et al..^15^ reported, when compared with knot-tying anchors, knotless anchors showed equal rates of re-dislocation and revision surgery, as well as reduced rates of recurrent subluxation. Patient functional results ranged from satisfactory to outstanding. This adds to the evidence that knotless anchors are equally effective as knot-tying anchors for the arthroscopic anterior labral repair of recurrent anterior shoulder dislocation. Knotless all-suture anchor repair saves time, reduces knot-tying irregularities, and removes knot stacking and the risk of knot abrasion, which can cause soft tissue or cartilage injury.^16^^,^^17^

Methods to prevent capsular–labral tissue tears and improve tissue-holding power were first described by Castagna et al.^18^ A lark’s head knot in the LL loop effectively provides lateral cut resistance, allowing the glenoid labrum complex to be anchored more firmly without the risk of tearing due to poor tissue quality. Combined with the aforementioned, we seem to have reason to believe that the technology should have superior advantages.

### Limitations

Specific experimental evidence regarding the height of the glenoid labrum, biomechanically, is not available and further experimental confirmation is needed. Although it has been demonstrated that proper tightening of the joint capsule does not affect joint mobility, the specifics need to be followed up with the patient on an ongoing basis.

## Conclusions

In general, the LL loop is a high-strength suture method that simultaneously tightens the shutter capsule in the direction of longitude and latitude and resists tearing with knotless. However, further biomechanical studies and clinical trials need to be carried out to evaluate the biomechanical properties of this Bankart repair and the clinical outcome of the recurrence rate.

## Supplementary Data

ICMJE author disclosure forms

Video 1 The longitude–latitude loop used for complex Bankart lesion repair. This is a right shoulder and the visualization starts in the posterior portal. Properly investigate the pathologic Bankart lesion. Thoroughly freshen the cartilage of the articular glenoid rim and the lateral bone bed of the articular glenoid. The area of injury is completely released to the medial subscapularis muscle belly with the rasp, and the glenoid labrum is loosened and floated off the articular surface. A lark’s head knot is constructed on the capsule–labrum complex tendon with a No. 2 FiberWire suture. One suture strand exits through the tissue where the labrum and glenoid tear. Create the longitude–latitude loop at 5:20-4:30, 4:20-3:30, and 3:20-2:20 o’clock and use different-colored FiberWire threads to separate multiple loops. According to the location of the longitude–latitude loop, drill a 2.9-mm hole 1 mm inside the 5:30-o’clock joint margin. FiberWire strands of the longitude–latitude loop are passed through the PushLock 2.9-mm anchors while the other FiberWires of each group are placed outside the anterior portal and the glenoid labrum of the joint capsule is lifted and repositioned. Tighten the upper end and the lower end in turn and tighten the screws, pushing up the glenoid labrum tissue to form the Bump. Fix all the longitude–latitude loops with 2.9-mm anchors, then cut off the residual suture, and a novel, self-locking, and high-resistant loop configuration is constructed.
